# *Helicobacter pylori* prevalence in the Southwest of China: A protocol for systematic review

**DOI:** 10.1097/MD.0000000000019369

**Published:** 2020-03-13

**Authors:** Rui Wang, Dan Bai, Wen Xiang, Yu-Feng Zhang, Kun-Yi Ba, Xin-Zu Chen

**Affiliations:** a Nursing Section, Department of Gastroenterology; b Department of Gastrointestinal Surgery and Laboratory of Gastric Cancer, West China Hospital; c Faculty of Clinical Medicine, West China Medical School, Sichuan University, Chengdu; d Department of Gastrointestinal and Hernia Surgery, The Second People's Hospital of Yibin, West China Yibin Hospital, Sichuan University, Yibin; e Department of General Surgery, The First People's Hospital of Longquanyi, West China Longquan Hospital, Sichuan University, Chengdu, China.

**Keywords:** eradication, gastric cancer prevention, *Helicobacter pylori*, prevalence, screening

## Abstract

**Objectives:**

This epidemiological research will be aimed to evaluate the longitudinal changes of *Helicobacter pylori* prevalence in Southwest China during recent period through a systematic review and analysis.

**Methods:**

The database PubMed and China National Knowledge Infrastructure will be searched. The cross-sectional studies or cohort studies on either massive or hospital-based health checkup population will be potentially eligible. The study population was originated from one of the southwestern major cities, Chengdu (Sichuan), Chongqing, Kunming (Yunnan), Guiyang (Guizhou), or Lhasa (Tibet). Two reviewers will independently select studies, extract data, and assess the quality of studies. The prevalence of *H pylori* infection will be estimated. In the individual city, the longitudinal comparisons will be conducted to evaluate the trends referring to the earliest cross-sectional baseline. The risk ratio and its 95% confidence interval will be estimated. Subgroup analyses will be performed in sex-specific and age-specific subsets. Trend analysis for proportions (p for trend) will be estimated in the longitudinal evaluation. If applicable, the longitudinal clearance rate (%) will be estimated.

**Ethics and Dissemination:**

The ethical approval is not required due to the nature of literature-based research. The results will be disseminated through meetings and a peer-reviewed journal.

**PROSPERO Registration Number:**

CRD42019120764

## 1 Introduction

Since 1994, the World Health Organization has defined *Helicobacter pylori* (Hp) as a class-I carcinogenic pathogen for gastric cancer.^[1]^ The infection of Hp was suggested as an important risk factor for gastric cancer in both western and eastern countries.^[2–4]^ Although gastric cancer was relatively rare in Germany, the strong association between Hp infection and gastric cancer risk was confirmed in a population-based longitudinal cohort.^[2]^ Massive Hp screening and eradication were organized in Japan and South Korea as a fundamental approach to identify high-risk subpopulation and prevent occurrence of disease.^[5]^ Understanding the prevalence of Hp might be helpful to population-tailored screening and eradication strategy to prevent gastric cancer.^[6]^

**Figure 1:**
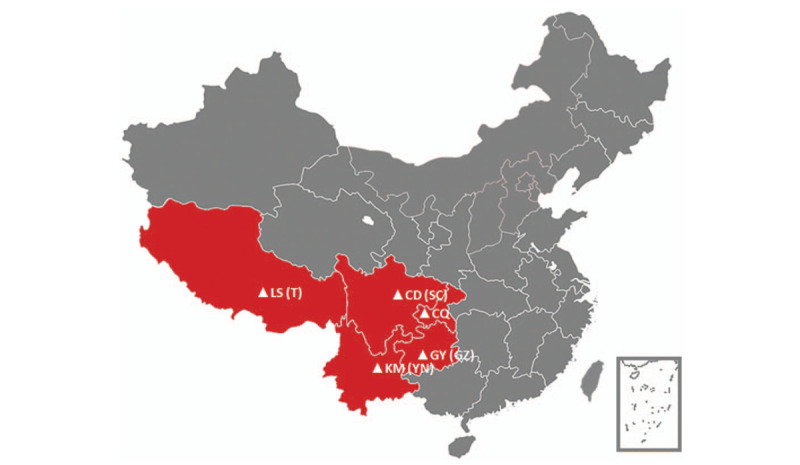
Major cities in Southwest China. CD = Chengdu, CQ = Chongqing, GY = Guiyang, GZ = Guizhou province, KM = Kunming, LS = Lhasa, SC = Sichuan province, T = Tibet, YN = Yunnan province.

The incidence of gastric cancer was fairly high in China,^[7]^ and led to heavy burden of high mortality due to low proportion of early diseases.^[8,9]^ Southwest China is a less developing region with multiple ethnics, accounting for 24.5% of China's land area and 14.1% of total population (Fig. 1). The infection of Hp was common in Southwest China, but organized screening of Hp and gastric cancer was not completely established yet. However, along with the updates and introduction of the serial Maastricht consensuses,^[10–12]^ the awareness and willingness to screening Hp became more popular in Southwest China. This epidemiological research will be aimed to evaluate the longitudinal changes of Hp prevalence in Southwest China during recent periods through a systematic review and analysis.

## 2 Methods

### 2.1 Literature search

The PubMed database will be searched up to December 31, 2019, with the search strategy “(“helicobacter pylori”[MeSH Terms] OR (“helicobacter”[All Fields] AND “pylori”[All Fields]) OR “helicobacter pylori”[All Fields]) AND (sichuan[All Fields] OR chengdu[All Fields] OR chongqing[All Fields] OR yunnan[All Fields] OR kunming[All Fields] OR guizhou[All Fields] OR guiyang[All Fields] OR tibet[All Fields] OR xizang[All Fields] OR lasa[All Fields] OR lhasa[All Fields] OR (west[All Fields] AND (“china”[MeSH Terms] OR “china”[All Fields])) OR (western[All Fields] AND (“china”[MeSH Terms] OR “china”[All Fields])) OR (southwest[All Fields] AND (“china”[MeSH Terms] OR “china”[All Fields])) OR (southwestern[All Fields] AND (“china”[MeSH Terms] OR “china”[All Fields]))).” Similarly, the literature search will be performed in the China National Knowledge Infrastructure database of Chinese academic journals through the similar strategy. Those nonjournal data of our institute West China Hospital, a central teaching hospital in Sichuan, will be additionally included. The publication language in English or Chinese will be acceptable.

### 2.2 Eligibility

The cross-sectional studies or cohort studies on either massive or hospital-based health-checkup population will be potentially eligible. Those studies which contained or were contaminated with gastric malignancies will be excluded. Those researches on healthy persons in an experimental setting instead of health checkup will be also excluded. The study population was originated from one of the southwestern major cities, Chengdu (Sichuan), Chongqing, Kunming (Yunnan), Guiyang (Guizhou), or Lhasa (Tibet). The prevalence of Hp infection was tested by any method including breath test (^13^C-urea or ^14^C-urea), or serological IgG test among observations. There should be no limitation in age, sex, and ethnics of observations.

### 2.3 Selection and quality assessment

The search results from the 2 databases will be combined by a reviewer, and then the duplicate literature will be eliminated. Two reviewers will separately browse the titles/abstracts and assess the potentially eligible full-texts, according to the predefined inclusion and exclusion criteria. Discrepancies will be resolved by consensus with a third reviewer. Risk of bias assessment of all included studies will be independently performed by 2 reviewers, according to the Newcastle-Ottawa Scale.^[13]^ The scale contains 8 criteria of 3 categories to evaluate the sample selection, comparability on the bases of design or analysis, outcome assessment.

### 2.4 Data extraction

The basic information of study city, cross-sectional or cohort baseline period, and test type will be recorded. The sample size and positive events of Hp infection will be extracted. Sex-specific and age-specific events and subtotals will be extracted additionally.

### 2.5 Statistics

The STATA 14.0 softwares will be used for statistical analysis. The prevalence (%) of Hp infection and its 95% confidence intervals will be estimated. In the individual city, the longitudinal comparisons will be conducted to evaluate the trends referring to the earliest cross-sectional baseline. The risk ratio and its 95% confidence intervals will be estimated. Subgroup analyses will be performed in sex-specific and age-specific subsets. Trend analysis for proportions (p for trend) will be estimated in the longitudinal evaluation. In addition, if applicable, the longitudinal clearance rate (%) will be estimated as Clearance (%) = [(prevalence_(*i*)_ − prevalence_(*j*)_)/prevalence_(*i*)_]%; (*i*) and (*j*) were sequential age groups with a 10-year interval.

### 2.6 Ethics

The ethical approval is not required due to the nature of literature-based research.

### 2.7 Registration

The present systematic review was registered in the PROSPERO International Prospective Register of Systematic Reviews supported by the National Institute for Health Research of the National Health Service, UK (registration number: CRD42019120764).^[14]^

### 2.8 Reporting

This systematic analysis will be conducted according to the MOOSE 2000 statements,^[15]^ and a flow diagram will be drawn.

### 2.9 Dissemination

The results will be disseminated through meetings and a peer-reviewed journal.

## 3 Discussion

Health burden resulted from digestive system cancers was still heavy in China, especially the gastric cancer–related high mortality.^[8,16]^ Because of the diversity of economics, ethnics, and lifestyles,^[17,18]^ it should be meaningful to investigate the tailored prevention and screening plan for cancers. As known, the Hp infection is an identified risk of gastric cancer.^[19,20]^ In addition, Hp infection might be an potential risk factor for the development of pancreatic or colorectal cancers.^[21,22]^ Therefore, the present study is aimed to observe and analyze the longitudinal changes of Hp prevalence in Southwest China during recent periods. The Sichuan Gastric Cancer Early Detection and Screening project was based on an investigator-driving group in West China Hospital of Sichuan University. Serial researches are conducted under the Sichuan Gastric Cancer Early Detection and Screening project to provide information for the tailored screening of gastric cancer in Sichuan province, even in Southwest China.^[17,18,23,24]^ For example, the prevalence of Hp was quite high up to >80% among Tibetans in decades ago,^[8]^ but the epidemiologic changes and status in recent years might be informative to design the Hp screening and eradication protocol in Tibetans. Optimal or cost-effective screening and eradication protocol is able to reduce the gastric cancer incidence and related mortality.^[25,26]^ Compared to the experiences of gastric cancer prevention and control in Japan and Korea,^[9,27–29]^ a lot of researches on etiology, high-risk population, screening, and surveillance protocol and economics are fairly required in China. The evidence-based conduction of gastric cancer prevention and control would be believably organized in the future.

## Acknowledgments

The work was based on the Sichuan Gastric Cancer Early Detection and Screening (SIGES) project. The authors thank the substantial work of Volunteer Team of Gastric Cancer Surgery (VOLTGA), West China Hospital, Sichuan University, China.

## Author contributions

Conception, registration, literature search, and research conduction: Rui Wang.

Literature selection, data extraction, and quality assessment: Dan Bai, Wen Xiang, Yu-Feng Zhang, Kun-Yi Ba.

Conception, analysis, interpretation, writing, and academic inspection: Xin-Zu Chen.
